# OA13 Diagnostic utility of vessel-wall MRI in giant cell arteritis-associated anterior ischaemic optic neuropathy

**DOI:** 10.1093/rap/rkag113.013

**Published:** 2026-11-03

**Authors:** Sarah Al-Saad, Afzal Latheef, James Cai, Dalia Ludwig

**Affiliations:** UCLH, London, United Kingdom; UCLH, London, United Kingdom; UCLH, London, United Kingdom; UCLH, London, United Kingdom

## Abstract

**Introduction:**

Giant cell arteritis (GCA) is a diagnostically challenging systemic vasculitis affecting large and medium vessels and is a medical emergency with devastating complications such as permanent blindness and stroke.

Diagnosis relies on clinical assessment, laboratory markers, imaging modalities and biopsy. BSR and EULAR guidelines recommend confirmatory testing with temporal artery ultrasound/biopsy as first line, with FDG-PET or MRI as alternative or complementary modalities.

This atypical case underlines the importance of high-resolution vessel-wall MRI when first-line vascular investigations are inconclusive and demonstrates the role of 3D vessel-wall imaging with collaborative MDT expertise in increasing diagnostic confidence and guiding treatment escalation.

**Case description:**

An 81-year-old non-smoking Caucasian woman was referred from neuro-ophthalmology clinic.

She had a 3-week history of viral-like illness with no jaw claudication, temporal symptoms or scalp tenderness but had frontal headache and diplopia, all of which self-resolved prior to assessment. During this period, she had three transient episodes (lasting seconds) of right-sided blurred vision.

Her medical history included hypertension, hypercholesterolaemia, rheumatoid arthritis and psoriasis.

Initial ophthalmic examination demonstrated reduced right visual acuity (6/12 with no improvement on pinhole and left eye 6/9 improving with pinhole), slow colour vision, right optic disc swelling with blurred margins and an inferior right visual field defect.

During rheumatology assessment, clinical examination was normal. (CRP 100 mg/L, ESR 116 mm/hr, WCC 10.6 x10^9^/L, platelets 521 x10^9^/L and haemoglobin of 112 g/L). Treatment was initiated as per BSR guidelines.

Bilateral temporal artery ultrasound was normal. Initial MRI head with contrast demonstrated a punctate area of restricted diffusion in the left forceps major, suggesting acute infarct, and mild right optic nerve intra-orbital segment perineural enhancement. PET-CT showed mildly increased mural uptake in the right vertebral artery (V3 segment). CT angiography of intracranial and neck vessels was normal.

Stroke MDT found insufficient imaging evidence to support vasculitis, with no suitable area for biopsy.

Due to persistent clinical suspicion of GCA, further vessel-wall MRI with contrast was arranged through large-vessel vasculitis MDT.

Dedicated imaging demonstrated pathological enhancement of both ophthalmic arteries, posterior ciliary arteries and periorbital fat near the globe bilaterally. It also demonstrated concentric vessel-wall enhancement of the vertebral arteries. These findings were consistent with GCA, supporting anterior ischaemic optic neuropathy (AION), with vertebral artery involvement causing posterior circulation infarct.

Treatment was escalated to tocilizumab due to further visual deterioration. Her GCA remains in remission currently with improved right optic disc swelling and field defect.

**Discussion:**

This case illustrates the diagnostic complexity when clinical features and conventional investigations are non-concordant. Arteritic anterior ischaemic optic neuropathy (A-AION) is mostly caused by GCA in elderly patients and distinguishing them from non-arteritic AION is extremely important to maximise functional prognosis.

First-line vascular investigations did not provide diagnostic certainty in this case. The temporal artery ultrasound was normal, FDG-PET demonstrated only mildly increased uptake in the right vertebral artery, raising clinical suspicion but not establishing a diagnosis.

This patient underwent dedicated vessel-wall MRI (vw-MRI) with contrast including whole-head intracranial time-of-flight angiography, volumetric FLAIR and 3D post-contrast vessel-wall imaging.

In this context, high-resolution black-blood MRI provided important evidence of disease in two clinically relevant territories: orbital arterial involvement affecting the ophthalmic and posterior ciliary arteries bilaterally, supporting A-AION; and vertebral artery involvement, providing a possible mechanism for the posterior circulation infarct.

High-resolution vw-MRI offers a comprehensive assessment of blood vessels, soft tissue and the orbits offering excellent diagnostic accuracy in vasculitis with sensitivity of 94% and 85% specificity in GCA and reduces need for temporal artery biopsy. Absence of vessel wall enhancement has a negative predictive value of 100% for temporal artery biopsy.

BSR and EULAR recommend exploring vessel wall imaging in similar situations. Recent publications recommend using a 3-Tesla scanner with minimum 16-channel head coil and high in plane resolution to achieve necessary spatial resolution whereas 3D whole brain sagittal or coronal black-blood imaging or 2D axial imaging are recommended for scalp vessels.

This imaging modality can help with risk stratification, identify patients at risk of contralateral AION, avoid overtreating mislabelled NA-AION patients and also increase physician confidence when initiating treatment.

**Key learning points:**

This diagnostically ambiguous case of GCA with visual involvement demonstrates how vessel-wall MRI can bridge the gap between clinical suspicion and diagnostic confidence. By localising vessel-wall inflammation across ocular and extracranial vascular territories, it helped unify the patient’s visual and neurovascular findings and supports the emerging role of vw-MRI in future diagnostic pathways.

1. High-resolution vessel-wall MRI may provide valuable diagnostic evidence in selected cases of atypical GCA.

2. Specialist MDT interpretation is central to the value of advanced vessel-wall imaging.

3. Further studies are needed to define standardised vessel-wall MRI protocols and scoring systems before integration into diagnostic pathways.

4. Vessel-wall MRI may have an expanding role in differentiating A-AION from NA-AION, monitoring disease activity and treatment response, and stratifying risk in complex GCA presentations.

